# Commentary: Profiling of UGT1A1^*^6, UGT1A1^*^60, UGT1A1^*^93, and UGT1A1^*^28 Polymorphisms in Indonesian Neonates With Hyperbilirubinemia Using Multiplex PCR Sequencing

**DOI:** 10.3389/fped.2019.00528

**Published:** 2020-02-07

**Authors:** Mohammad Reza Heydari, Majid Fardaei

**Affiliations:** ^1^Shiraz HIV/AIDS Research Center, Institute of Health, Shiraz University of Medical Sciences, Shiraz, Iran; ^2^Department of Medical Genetic, Medical School, Shiraz University of Medical Sciences, Shiraz, Iran

**Keywords:** linkage disequilibrium, hyperbilirubinemia, neonatal, UGT1A1 enzyme, Gilbert disease

Neonatal hyperbilirubinemia is a common benign phenomenon, related to a variety of physiological, pathological, and genetic conditions of neonates (1). Since this disorder can lead to neurodevelopmental impairment, finding the predictable factors could guide clinicians to provide better care (2). We read this article by Amandito et al. and congratulate the authors and also provide some suggestions.

In this well-designed cross-sectional study, the genetic sequencing was fully performed in different parts of the UGT1A1 gene, which is related to Gilbert syndrome (3). Finally, the researchers made an attempt to create a link between a neonate's genetic map with his/her bilirubin level. In this study, four polymorphisms in the UGT1A1 gene were investigated. By paying attention to the location of the single nucleotide polymorphisms, at least two genes UGT1A1^*^60(−3279T>G) and UGT1A1^*^6(−3156G>A) are situated in very close proximity (4).

An important concept in genetic polymorphism is linkage disequilibrium. It means that two genes are physically linked to each other, and alleles do not occur randomly with respect to each other (5).

In conclusion, due to the high likelihood of these two mutations moving together (6), the researchers have to calculate the linkage disequilibrium value before performing the statistical tests. If the *D*′ value was high, in their analysis, two mutations were considered as one. For this reason, they have to decide to test this mutation together either or alone.

## Author Contributions

All authors listed have made a substantial, direct and intellectual contribution to the work, and approved it for publication.

### Conflict of Interest

The authors declare that the research was conducted in the absence of any commercial or financial relationships that could be construed as a potential conflict of interest.
